# Analysis of the therapeutic efficacy of robot-assisted percutaneous screw fixation in the minimally invasive treatment of pelvic fractures

**DOI:** 10.3389/fsurg.2024.1392719

**Published:** 2024-07-03

**Authors:** Mige Wang, Song Zheng, Yushan Zhang, Jialing Lu

**Affiliations:** Department of Traumatic and Pediatric Orthopedics, The Second Affiliated Hospital of Jiaxing University, Jiaxing, Zhejiang, China

**Keywords:** orthopedic robot, pelvic fracture, percutaneous screws, minimally invasive operation, surgical robot

## Abstract

**Objective:**

To compare the therapeutic efficacy of robot-assisted and manual screw placement techniques for the treatment of pelvic fractures.

**Methods:**

This study included patients with pelvic fractures admitted to our orthopedic department between January 2020 and January 2022. They were randomly assigned to either the robot-assisted group or the control group. Various parameters, including surgical duration, intraoperative bleeding, fluoroscopy frequency, postoperative pain, length of hospitalization, postoperative hematological indices, postoperative functional scores, and postoperative complications, were compared between the two groups.

**Results:**

There were no significant differences in age, sex, body mass index, and preoperative hematological parameters between the two groups. The robot-assisted group exhibited significantly shorter surgical duration, lower fluoroscopy frequencies, lower postoperative pain scores, and shorter length of hospitalization compared to the control group. At 3 and 6 months postoperatively, patients in the robot-assisted group demonstrated significantly higher Majeed functional scores in comparison to the control group. However, there were no significant differences in Majeed scores at 12 months postoperatively. Moreover, there were no significant differences in postoperative complications between the two groups.

**Conclusion:**

Robot-assisted minimally invasive treatment of pelvic fractures using hollow screws effectively reduced surgical duration, mitigated intraoperative bleeding and postoperative pain, shortened hospital stays, and promoted faster functional recovery.

## 1 Introduction

Pelvic fractures, which occur as a result of high-energy trauma, constitute approximately 3%∼8% of all fractures and are associated with a mortality and disability rate as high as 18 % (1, 2). The pelvic structure is intricate, housing vital blood vessels, nerves, and organs nearby, thus rendering conventional open surgery a risky endeavor. While percutaneous screw techniques have mitigated surgical risks for certain cases, their success still hinges on the surgeon's expertise and carries the potential for damaging adjacent tissues during screw implantation.

The advent of computer technology and artificial intelligence in the medical field has facilitated the introduction of robotic navigation and precise minimally invasive treatment techniques in orthopedics. By employing surgical robots to expedite planning and navigate the screw trajectory with utmost precision, intraoperative complications can be effectively reduced (3, 4). This study aims to compare the use of robot-assisted guidance and conventional C-arm guidance in the minimally invasive treatment of pelvic fractures, thereby exploring the practical application of robots in the realm of pelvic fracture management.

## 2 Materials and methods

### 2.1 General information

Data was collected from patients admitted to our orthopedic department with pelvic fractures between January 2020 and January 2022. The inclusion criteria encompassed the followings: (1) Patients with recent closed pelvic fractures; (2) Patients aged between 20 and 70 years; (3) Fractures devoid of displacement or exhibiting mild displacement that met the prerequisites for screw placement after preoperative traction, closed reduction, or limited open reduction. On the other hand, the exclusion criteria entailed: (1) Patients with open or dated pelvic and acetabular fractures; (2) Patients with concurrent spinal cord, cranial, or peripheral vascular and nerve injuries in the pelvic region; (3) Patients with severe medical conditions that rendered them unsuitable for surgery; (4) Patients with severe osteoporosis. It's defined that patients' dual-energy x-ray *T*-score were < −2.5; (5) Patients with pubic symphysis diastasis, isolated posterior wall fractures of the acetabulum, significantly displaced posterior column fractures or displaced posterior column posterior wall fractures.

The eligible patients were randomly allocated to either the robot-assisted group or the control group (C-arm group) via sealed envelope randomization. This study received approval from the Ethics Committee of the Second Affiliated Hospital of Jiaxing University (2020JX079). Before the commencement of surgery, the patients were provided with comprehensive explanations regarding their conditions and the associated risks of participating in the study. Written informed consent was obtained from all patients.

### 2.2 Surgical methods

**Figure 1:**
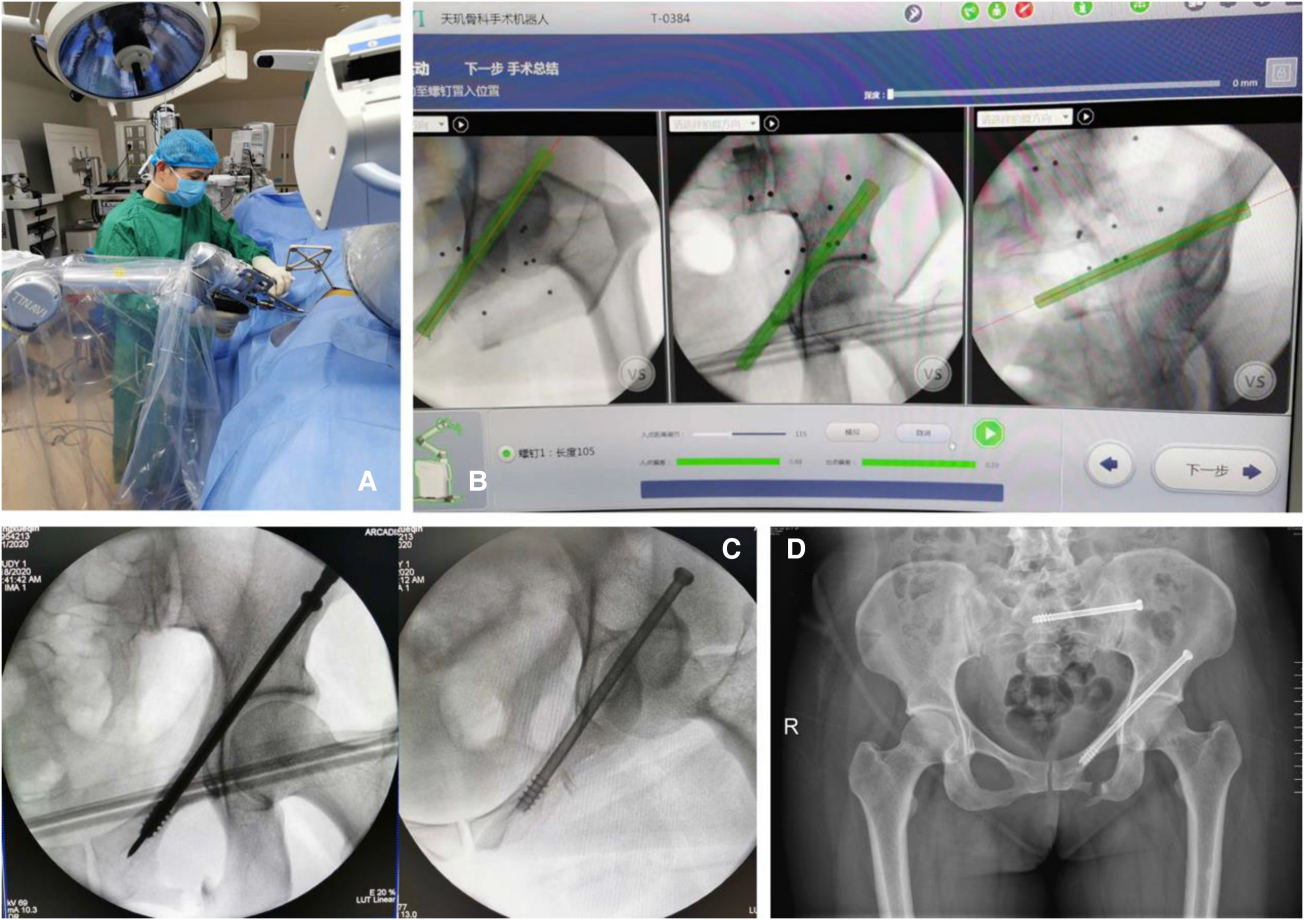
(**A**) Intraoperative manipulation of the surgical robot; (**B**) computer-generated planning image during the surgery; (**C**) guide wire for the percutaneous insertion of hollow screws guided by computer planning and the surgical robot; (**D**) postoperative x-ray photo.

**Figure 2:**
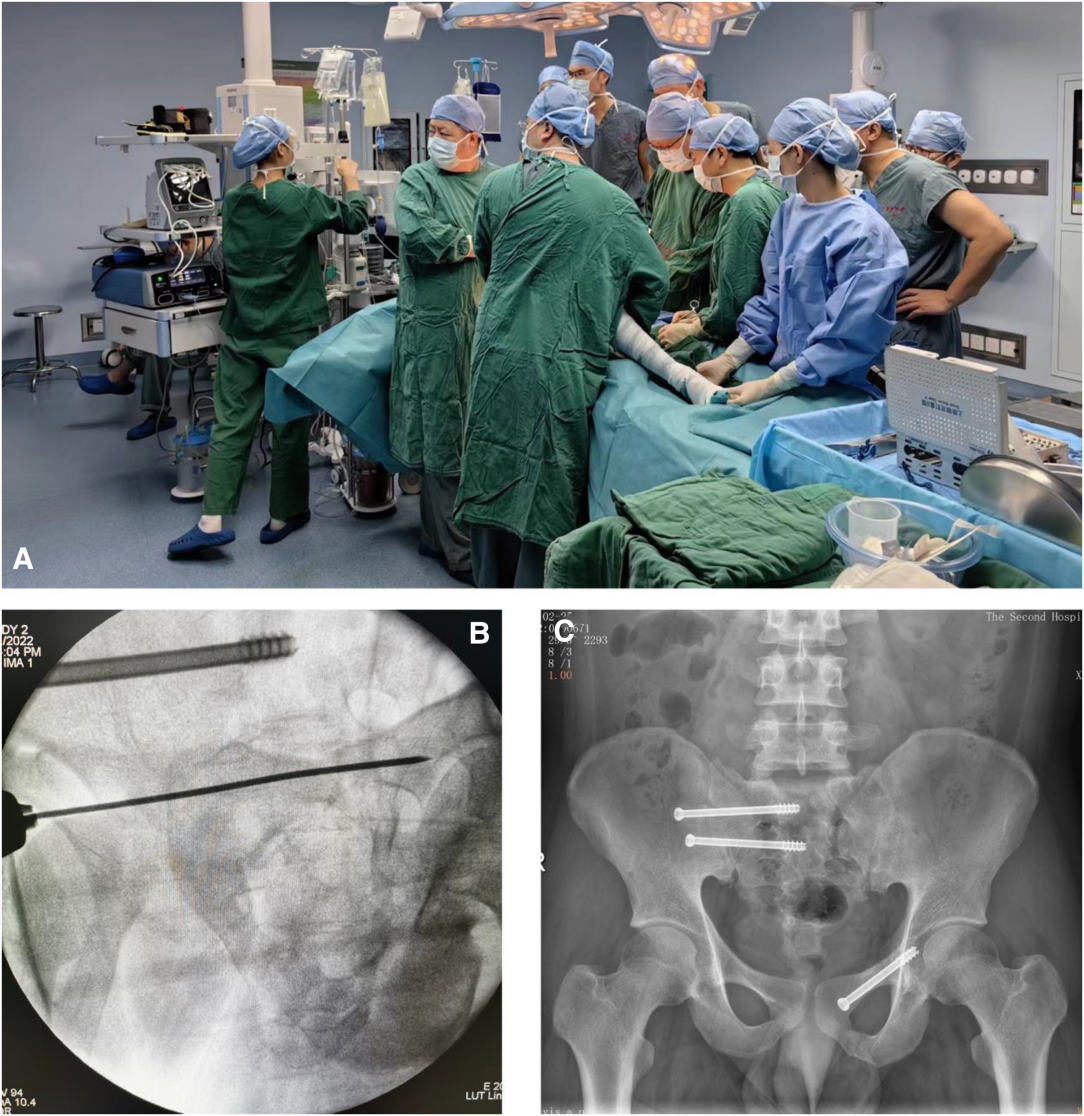
(**A**) Intraoperative manipulation manually in control group; (**B**) guide wire for the percutaneous insertion of hollow screws guided by C-arm; (**C**) postoperative x-ray photo.

Both groups underwent surgical procedures conducted by the same team. In the robot-assisted group, the Tinavi^TM^ orthopedic surgical robot was employed for assistance. Intraoperatively, a C-arm x-ray machine and an optical tracking camera were used for positioning and image acquisition. These images were subsequently imported into the Tinavi™ system for planning purposes. Once the planning phase was completed, the robotic arm autonomously moved to the entry point, allowing for the percutaneous insertion of the guide wire into the fracture site via the sleeve on the robotic arm. Following fluoroscopic verification, the hollow screw was inserted into the body along the guide wire (Figure 1). Conversely, in the control group, the guide wire was manually drilled into the fracture site under the monitoring of the C-arm x-ray machine. After fluoroscopic verification, the hollow screw was inserted into the body along the guide wire (Figure 2).

Postoperatively, prophylactic antibiotics were administered to prevent infection. Bedside functional exercises were guided on the second day following surgery, and crutch walking commenced at 6–8 weeks postoperatively. At 10–12 weeks postoperatively, radiographic reexamination was conducted and full-weight bearing walking was initiated.

### 2.3 Measures and evaluation of outcomes

We collected preoperative characteristics including age, sex, body mass index (BMI), and preoperative hematological indicators. Intraoperative conditions, such as surgical duration, intraoperative bleeding, and fluoroscopy frequency, were also recorded. Additionally, postoperative conditions, including postoperative hospitalization duration, pain score, postoperative hematological indicators, postoperative complications, and functional scores at 3, 6, and 12 months postoperatively, were assessed.

Pain score was evaluated using the Visual Analog Scale (VAS), while postoperative function was measured using the Majeed Score (5). The positioning of the screws was analyzed by post-operative CT scan and was categorized as follows: “optimal” if the screw was entirely within the channel, “good” if the screw made partial contact with the cortex without perforation, and “poor” if the screw perforated the cortex.

### 2.4 Statistical analysis

Statistical analyses were performed using IBM SPSS Statistics software (version 23.0). Student's *t*-test was used for continuous variables, and the chi-square test was used for categorical variables. A *p*-value < 0.05 was considered statistically significant.

## 3 Results

**Figure 3:**
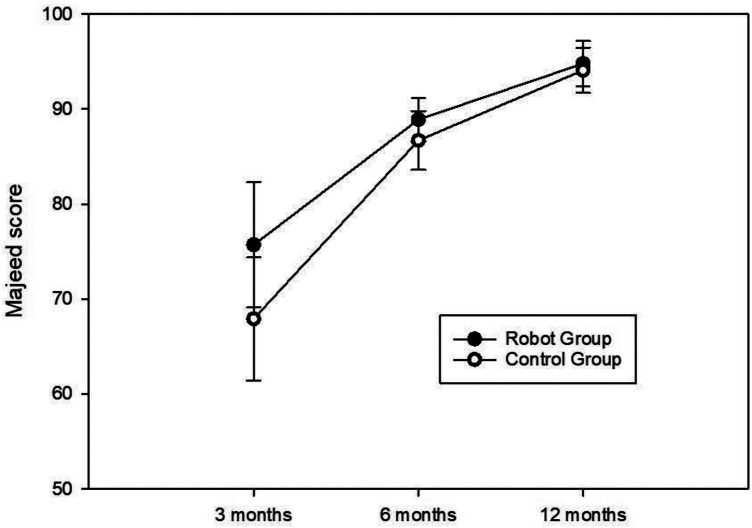
The trajectory of majeed scores at 3, 6, and 12 months postoperatively in the two patient groups.

A total of 55 patients diagnosed with pelvic fractures were enrolled in this study, comprising 34 males and 21 females, with a mean age of 54.7 ± 17.8 years. There were no significant differences observed in age, sex, BMI, and preoperative hemoglobin, albumin, and white blood cell (WBC) count between the two groups (Table 1). The robot-assisted group demonstrated a significantly shorter surgery duration and a reduced number of fluoroscopies compared to the control group, while intraoperative bleeding displayed no statistically significant difference between the two groups (Table 2). Moreover, the robot-assisted group exhibited lower postoperative pain scores and shorter hospitalization in comparison to the control group. However, there were no differences in postoperative hematological indicators (Table 3). At 3 and 6 months postoperatively, the robot-assisted group exhibited significantly higher Majeed functional scores than the control group. Nevertheless, there was no significant difference in Majeed score at the 12-month follow-up (Table 4, Figure 3). Except for one patient in each group with a good screw position grade, the remaining patients in both groups achieved an optimal screw position. Notably, one patient in each group developed deep vein thrombosis postoperatively, without symptoms of pulmonary embolism. Furthermore, both groups recorded no cases of incision infection, poor wound healing, or internal fixation failure as postoperative complications.

**Table 1:** General characteristics of patients in the two groups.

	Robot-assisted group	Control group	*P*-value
Case size	26	29	–
Sex (male/female)	13/13	21/8	0.153
Age, years	51.2 ± 18.1	57.9 ± 15.2	0.143
BMI, kg/m^2^	24.0 ± 2.4	23.9 ± 3.6	0.921
Hemoglobin, g/L	104 ± 20	113 ± 16	0.076
WBC, × 10^9^/L	8.4 ± 3.5	8.6 ± 3.1	0.872
Albumin, g/L	33.7 ± 3.3	35.4 ± 3.3	0.058

**Table 2:** Intraoperative conditions of patients in the two groups.

	Robot-assisted group	Control group	*P*-value
Surgical duration, minute	187 ± 55	231 ± 87	0.030
Intraoperative bleeding, ml	32 ± 18	48 ± 51	0.119
Fluoroscopy frequency	13 ± 2	22 ± 7	<0.001

**Table 3:** Postoperative conditions of patients in the two groups.

	Robot-assisted group	Control group	*P*-value
VAS score	2.9 ± 0.7	3.3 ± 1.0	0.034
postoperative hospitalization, day	9.1 ± 3.2	13.7 ± 10.8	0.043
Hemoglobin, g/L	96 ± 17	102 ± 10	0.079
WBC, ×10^9^/L	10.0 ± 3.6	11.5 ± 3.9	0.147
Albumin, g/L	31.9 ± 13.4	30.0 ± 3.3	0.483

**Table 4:** Postoperative majeed functional scores of patients in the two groups.

	Robot-assisted group	Control group	*P*-value
3 months postoperatively	75.7 ± 6.6	67.9 ± 6.5	<0.001
6 months postoperatively	88.9 ± 2.3	86.7 ± 3.1	0.006
12 months postoperatively	94.8 ± 2.4	94.1 ± 2.4	0.258

## 4 Discussion

Since the report by Matta and Saucedo in 1989 elucidating the concept of minimally invasive treatment through sacroiliac joint screws for posterior pelvic ring fractures (6), this technique has progressively garnered recognition. Consequently, exploration into the feasibility of percutaneous screw fixation for other bony channels within the pelvis has commenced. Given the intricate anatomical structure of the pelvis and its involvement in vital neurovascular structures, the safety margin for bony channels remains highly narrow. Percutaneous screw fixation necessitates a remarkable level of precision in terms of screw placement direction and angle (7). Historically, only experienced orthopedic surgeons possessed the proficiency to perform such procedures.

Computer-assisted technology has witnessed an escalating adoption in orthopedic surgery since the 1990s (8), particularly in the realm of percutaneous screw fixation for pelvic fractures. Navigation systems have undergone continuous refinement, with intraoperative computed tomography (CT) and magnetic resonance imaging navigation systems currently available. In recent years, computer-assisted optical positioning has also emerged as a novel approach in the domain of minimally invasive pelvic fracture treatment (9). According to the analysis conducted by Zwingman et al., the misplacement rate associated with various navigation techniques for screw insertion ranges from 0.1% to 1.3%, whereas the misplacement rate under traditional x-ray fluoroscopy stands at 2.6% (10, 11). The TiNavi^TM^ third-generation orthopedic surgical robot (known as TiRobot), deployed in this study, employs precise positioning through x-ray/CT and optical positioning devices to mitigate the misplacement rate during screw implantation.

Robotic technology facilitates the pre-planning of the optimal trajectory in a computerized environment before screw placement, obviating the need for the trial-and-error methodology inherent in manual screw insertion. Consequently, this approach reduces surgical time and intraoperative fluoroscopy, enhancing procedural efficiency. A recent meta-analysis involving 294 patients yielded compelling evidence that robot-assisted internal fixation engendered a noteworthy reduction in average surgical duration by 24 min and average fluoroscopy frequency by 2 times (12). In our study, the robot-assisted group evinced a substantial 20% reduction in surgical time compared to the control group, alongside a remarkable 40% decrease in fluoroscopy frequency, resulting in reduced iatrogenic injury to the patients during the procedure. Moreover, the robot-assisted group in this study demonstrated superior postoperative pain management relative to the control group, which potentially be attributed to the diminished intraoperative puncture-induced damage to the pelvis and surrounding soft tissues (13). Nonetheless, it is worth considering the marginal 0.4 difference in visual analog scale (VAS) pain scores between the two groups, prompting further exploration into the significance of this subjective perception.

The question surrounding the potential of robot-assisted surgery to enhance patient prognosis remains a topic of debate, as most studies have not observed discernible disparities in postoperative functional scores among patients (14). However, in this study, the robot-assisted group exhibited superior Majeed scores compared to the control group at 3 and 6 months postoperatively, plausibly attributable to the patients experiencing milder postoperative pain and shorter hospital stays. Nonetheless, the difference in functional scores between 3 and 6 months postoperatively has significantly decreased, and by 1 year postoperatively, the disparity between the two groups was eradicated. Hence, our study suggested that while robots might facilitate expedited functional recovery in patients, they did not ultimately improve prognosis. In contrast to conventional manual screw placement techniques, the critical significance of robot-assisted screw placement lies in the decentralization of surgical expertise. Previously, the hollow screw internal fixation for pelvic fractures often necessitated the involvement of seasoned senior surgeons. However, meticulous preoperative and intraoperative planning using computerized systems and precise guidance from robotic arms now enable the standardization of surgical procedures, empowering even younger surgeons to successfully undertake such surgeries (15).

This study does have certain limitations. The sample size is relatively small, and certain outcome measures may inadequately capture their differences. Furthermore, the follow-up duration is relatively short, lacking data from previous follow-ups. These limitations necessitate redress in future prospective studies boasting larger sample sizes and longer follow-up durations.

## 5 Conclusion

The utilization of robot-assisted minimally invasive interventions for pelvic fractures, involving hollow screws holds the potential to expedite surgical procedures, mitigate intraoperative bleeding and postoperative pain, abbreviate hospitalization stays, and facilitate swifter functional recuperation. Consequently, advocating for the widespread adoption of this approach is warranted.

## Data Availability

The original contributions presented in the study are included in the article/Supplementary Material, further inquiries can be directed to the corresponding author.
